# Taurine Provides Neuroprotection against Retinal Ganglion Cell Degeneration

**DOI:** 10.1371/journal.pone.0042017

**Published:** 2012-10-24

**Authors:** Nicolas Froger, Lucia Cadetti, Henri Lorach, Joao Martins, Alexis-Pierre Bemelmans, Elisabeth Dubus, Julie Degardin, Dorothée Pain, Valérie Forster, Laurent Chicaud, Ivana Ivkovic, Manuel Simonutti, Stéphane Fouquet, Firas Jammoul, Thierry Léveillard, Ryad Benosman, José-Alain Sahel, Serge Picaud

**Affiliations:** 1 Institut National de la Santé et de la Recherche Médicale UMR 968, Institut de la Vision, Paris, France; 2 L’université Pierre et Marie Curie, Université Paris 06, UMR_S 968, Institut de la Vision, Paris, France; 3 Centre National de la Recherche Scientifique UMR7210, Institut de la Vision, Paris, France; 4 Centre Hospitalier National d’Ophtalmologie des Quinze-Vingts, Paris, France; 5 Institut des systèmes intelligents et robotique, l’université Pierre et Marie Curie, Paris, France; 6 Centre National de la Recherche Scientifique UMR7222, Paris, France; 7 Institute of Ophthalmology, University College of London, London, United Kingdom; 8 Fondation Ophtalmologique Adolphe de Rothschild, Paris, France; 9 French Academy of Sciences, Paris, France; Faculty of Medicine University of Leipzig, Germany

## Abstract

Retinal ganglion cell (RGC) degeneration occurs in numerous retinal diseases leading to blindness, either as a primary process like in glaucoma, or secondary to photoreceptor loss. However, no commercial drug is yet directly targeting RGCs for their neuroprotection. In the 70s, taurine, a small sulfonic acid provided by nutrition, was found to be essential for the survival of photoreceptors, but this dependence was not related to any retinal disease. More recently, taurine deprivation was incriminated in the retinal toxicity of an antiepileptic drug. We demonstrate here that taurine can improve RGC survival in culture or in different animal models of RGC degeneration. Taurine effect on RGC survival was assessed *in vitro* on primary pure RCG cultures under serum-deprivation conditions, and on NMDA-treated retinal explants from adult rats. *In vivo*, taurine was administered through the drinking water in two glaucomatous animal models (DBA/2J mice and rats with vein occlusion) and in a model of *Retinitis pigmentosa* with secondary RGC degeneration (P23H rats). After a 6-day incubation, 1 mM taurine significantly enhanced RGCs survival (+68%), whereas control RGCs were cultured in a taurine-free medium, containing all natural amino-acids. This effect was found to rely on taurine-uptake by RGCs. Furthermore taurine (1 mM) partly prevented NMDA-induced RGC excitotoxicity. Finally, taurine supplementation increased RGC densities both in DBA/2J mice, in rats with vein occlusion and in P23H rats by contrast to controls drinking taurine-free water. This study indicates that enriched taurine nutrition can directly promote RGC survival through RGC intracellular pathways. It provides evidence that taurine can positively interfere with retinal degenerative diseases.

## Introduction

Taurine is a free amino-sulfonic acid mainly provided by nutrition, which is present in large amounts in the central nervous system, and in the retina where it represents nearly half of the free amino-acid content [1]. Although more than 30 years ago, taurine depletion was found to trigger photoreceptor degeneration in cats fed with a taurine-free diet [2], the mechanism of this taurine dependence still remains enigmatic. The effect of taurine on photoreceptor survival was subsequently confirmed in monkeys [3], in rats by administering an inhibitor or competitive substrate of the taurine transporter (Tau-T) [4] and finally achieved in mice by knocking-out the taurine transporter [5]. Taurine also appeared to play a major role in photoreceptor development [6], [7]. Although many studies investigated if taurine depletion could explain photoreceptor degeneration in different animal models of retinal diseases, a clear-cut link was never demonstrated [8]. However, a clinical study associating taurine, a calcium channel blocker (diltiazem) and vitamin E was reported to improve vision in patients with *Retinitis pigmentosa* [9]. Since these early discoveries involving taurine in photoreceptor development and survival, taurine was found to prevent neuronal excitotoxicty by reducing the glutamate-induced increase in intracellular calcium and endoplasmic reticulum stress [10], [11]. However, no clear taurine neuroprotective effect has been demonstrated in cerebral ischemia [12].

Recently, we demonstrated that the retinal toxicity of the antiepileptic drug vigabatrin, is caused by taurine depletion [13]. Vigabatrin-treated rats and mice were found to exhibit lower plasmatic taurine concentration and taurine supplementation decreased the observed photoreceptor degeneration and the consecutive disorganization of the photoreceptor layers. However, in vigabatrin-treated patients, the visual constriction is not only attributed to photoreceptor degeneration but also to retinal ganglion cell (RGC) loss; the RGC layer even appears as the primary site of retinal damage [14]–[17]. As for photoreceptors, taurine supplementation prevented vigabatrin-induced RGC degeneration [13], [18]. Finally, clinical relevance of these findings was obtained in toddlers treated for infantile spasms as they exhibited decreased plasmatic taurine concentrations [13]. However, as in taurine-depleted rat pups [19], it remained uncertain whether the RGC degeneration was a consequence of the ongoing degenerative process in photoreceptors or to a direct taurine-dependent effect on RGCs. In the meantime, experiments on a RGC immortalized cell line supported the idea that taurine could directly affect RGC survival [20].

To further investigate this hypothesis, we have examined the effect of taurine on purified adult rat RGCs in culture, on RGCs subjected to the selective N-Methyl D-Aspartate (NMDA)-induced excitotoxicity in retinal explants and on RGCs undergoing degenerative processes in several animal models of retinal diseases.

## Results

### Taurine Improves the Survival of Purified RGCs in Culture

**Figure 1:**
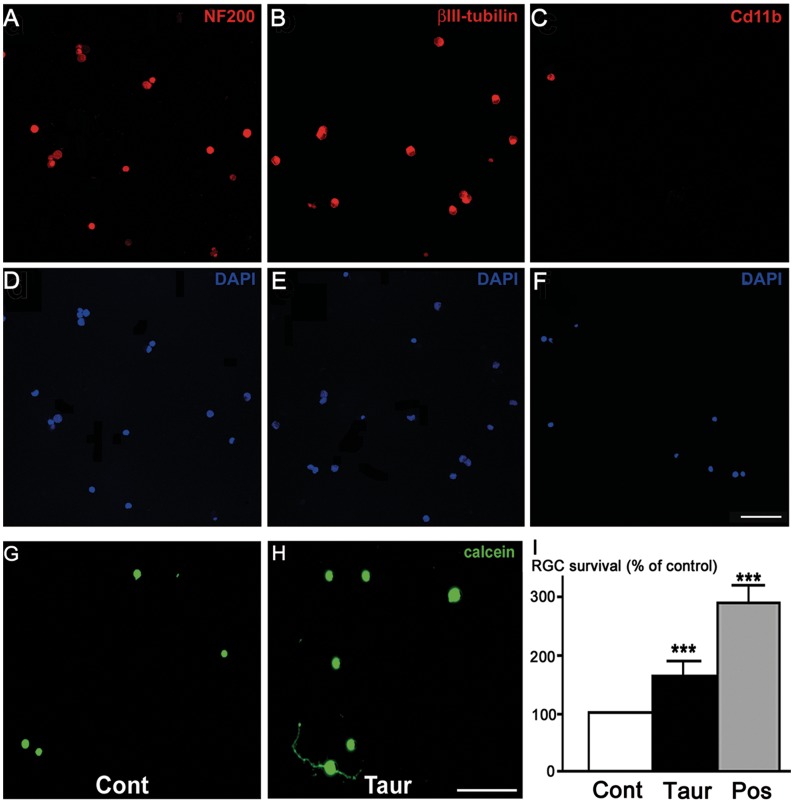
Taurine stimulates the survival of pure adult retinal ganglion cells (RGCs) in culture. A–F) Purity of RGC cultures. Confocal representative images of pure RGC cultures immunolabeled with specific RGC markers, NF-200 (red in A) or βIII-Tubulin (red in B), as well as with a specific marker for microglia/macrophages, Cd11b/c (red in C), while all isolated cells were stained by the nuclear dye DAPI (blue in D–F). Note that most cells were immunolabeled with the RGC markers (NF-200 or βIII-Tubulin) (A,B,D,E) whereas a very few cells were positive for the macrophage marker (C,F). G–H) Effect of taurine on pure RGCs. Representative images showing viable cultured RGC labeled with calceinAM, after 6 days *in vitro* (6 DIV), in the negative control condition (Cont; G) and following 1 mM taurine application (Taur; H). I) Quantification of RGC densities after 6 DIV either in the control condition (Cont; white bar), with 1 mM taurine application (Taur; black bar), or with the B27 supplement, providing a positive control condition (Pos; grey bar). In each experiment, the respective RGC densities were expressed as a percentage of the negative control condition at 6 DIV. Illustrated data are means ± s.e.m. from 21 independent experiments. ***p<0.001, one-way ANOVA followed by a Dunns post-hoc test. Scale bars represent 100 µm in panels (A–H).

To determine if taurine can affect directly RGC survival, it was applied on pure adult rat RGCs in the culture medium. The cell purity of adult RGC culture was first assessed by immunolabeling the RGC culture with two specific markers, NF200 and βIII-tubulin, or a marker for microglial cells and macrophages, Cd11b/c (Fig. 1A–F). Cell counting of these immunolabeling indicated that RGC purity in these cultures reached 98.0%±0.6% (n = 4) for NF-200 staining and 92.6±1.6% (n = 4) for βIII-Tubulin whereas Cd11b revealed very few microglial cells per field (Fig. 1C, F). To assess the protective effect of taurine, RGCs were cultured in a serum deprivation condition (low-nutritive medium composed by Neurobasal-A plus glutamine) that resulted in a low density of viable RGCs (3.3±0.3 RGC per field, n = 21; see Fig. 1G) when viability was revealed by calceinAM dye. A positive control was obtained by adding the B27 supplement that increased RGC survival by 190% (Fig. 1I). Interestingly, direct application of 1 mM of taurine into the culture medium for 6 days *in vitro* (DIV) significantly increased by 68% the RGC survival, as compared to RGCs cultured in taurine-free medium containing all twenty amino-acids, taken as control (Fig. 1H–I; p<0.001).

**Figure 2:**
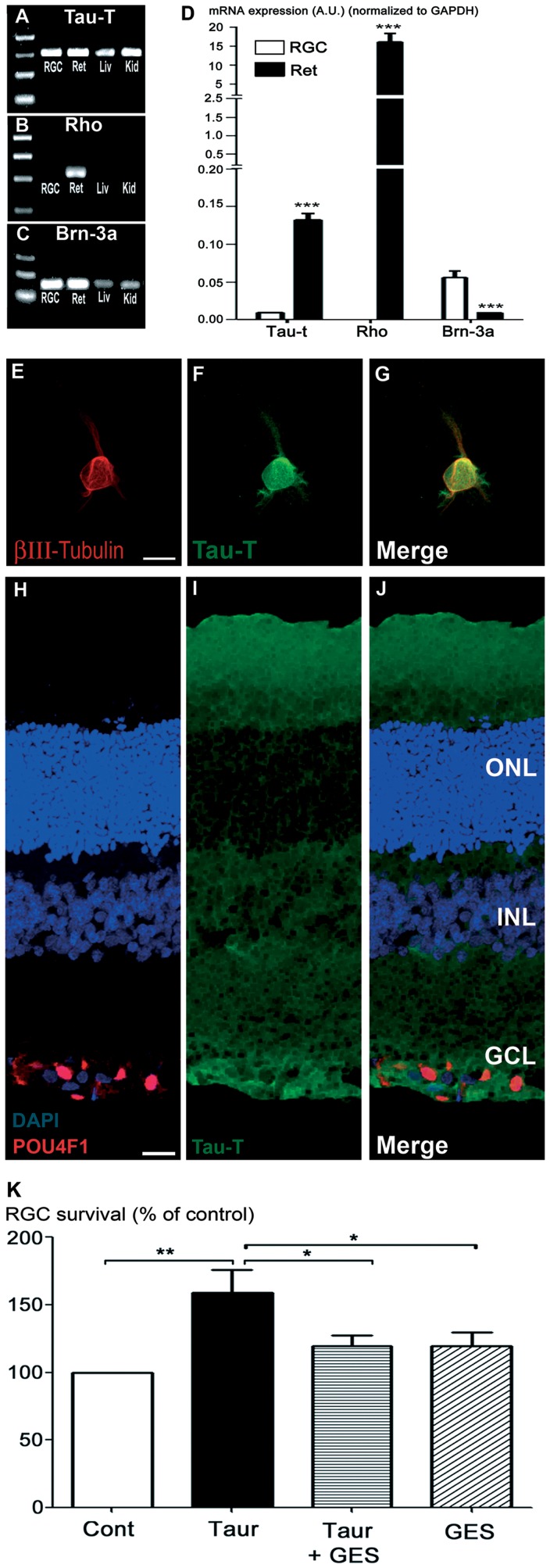
Implication of the taurine transporter in retinal ganglion cell (RGC) survival. A–C) Gene amplification (PCR) of the taurine transporter (Tau-T; A), rhodopsin (Rho; B) and POU4F1 (Brn-3a; C) performed on total cDNAs, previously obtained through a reverse transcription of total RNA extracted from freshly purified rat RGC (not cultured), rat full retina (Ret), rat liver (Liv), or rat kidney (Kid). Tau-T gene amplification revealed a high expression in pure RGCs (A). The purity of the RGC preparation was indicated by the high level of PCR products for POU4F1 (Brn-3a), a specific RGC transcription factor in the retina (Brn-3a in C) whereas no cDNA amplification was obtained for Rhodopsin (Rho), a major rod photoreceptor marker (Rho in B). D) Quantification by qPCR of mRNA expression for Brn3a, Rhodopsin (Rho) and the taurine transporter (Tau-T) in freshly purified RGCs (RGC, white bars) compared to the entire retina (Ret, black bars) from Long-Evans rats. The absence of rhodopsin cDNA amplification indicated that the taurine transporter expression in freshly purified RGCs (not cultured) cannot be attributed to a rod photoreceptor contamination. Results, normalized to GAPDH expression and presented as arbitrary units (A.U.), are means ± s.e.m. from 4 independent experiments for each gene tested. ***p<0.001 Ret groups compared to RGC groups, Two-tailed Student’s t-test. E–G) Representative confocal images showing Tau-T immunolabelling (green; F) in βIII-tubulin positive RGCs (red; E) after 6 days in culture. H–J) Representative confocal image of rat retinal sections immunolabelled with POU4F1 (red; H) and Tau-T (green; I) antibodies to illustrate localization of Tau-T in the ganglion cell layer (merge; J). K) Suppression of the taurine-elicited RGC survival by the taurine uptake inhibitor, GuanidinoEthane Sulfonate (GES). Quantification of RGC densities after 6 DIV either in control conditions (Cont, n = 11; white bar), in the presence of 1 mM taurine (Taur, n = 10; black bar), in the presence of 1 mM GES (GES, n = 9; oblique hatched bar) or in the presence of both taurine and GES (Taur+GES, n = 11; horizontal hatched bar). Illustrated data are means ± s.e.m. from independent experiments. ONL: outer nuclear layer; INL: Inner nuclear layer; GCL: Ganglion cell layer. ***p<0.001, **p<0.01 and *p<0.05, one-way ANOVA followed by a Dunns post-hoc test. Scale bars represent 10 µm in panels (E–G) and 20 µm in panels (H–J).

Since taurine is a potent antioxidant and an osmotic regulation molecule that requires Na^+^-dependent uptake to exert its cellular activities, we investigated whether RGCs express the taurine transporter (Tau-T). RGCs were purified and used immediately after the purification step (no culture) to measure the Tau-T expression level in freshly purified RGCs. As illustrated in Figure 2A, expression of the Tau-T was detected in these freshly purified adult rat RGCs. The RGC expression of Tau-T was not due to a photoreceptor contamination because our validation step demonstrated no rhodopsin expression in the purified RGC extracts (Fig. 2B, D), whereas these RGCs contained a high expression level of the transcription factor POU4F1 (Brn3a), RGC specific in the retina (Fig. 2C, D). The expression of Tau-T was also assessed by immunostaining in cultured RGCs. Figures 2E–G illustrates that all βIII-tubulin positive RGCs expressed Tau-T protein after 6 days in vitro (DIV) (n = 3 independent cultures, not shown). In addition, when rat retinal sections were immunolabeled with an antibody specific for Tau-T, the staining appeared particularly abundant in the ganglion cell layer (GCL) (Fig. 2I). These data indicate that RGC could generate taurine uptake both *in vitro* and *in vivo*. To assess if taurine uptake could account for the increase in RGC survival, taurine (1 mM) was co-incubated for 6 DIV with a blocker of the taurine transporter, GuanidinoEthane Sulfonate (GES, 1 mM) [4], in RGC cultures. Interestingly, addition of GES (1 mM) with taurine (1 mM) significantly reversed the protective effect exerted by taurine (+59%) on pure RGC cultures (p<0.05, Fig. 2F). In this condition, the difference in RGC survival was no longer statistically significant from the control condition (+19%, p>0.05). Similarly, application of GES alone (1 mM) did not significantly modify RGC survival as compared to control conditions (p>0.05, Fig. 2E). These results indicated that the protective effect of taurine on RGCs is critically dependent on the taurine transporter activity.

### Taurine Reduced RGC Death in NMDA-treated Retinal Explant

**Figure 3:**
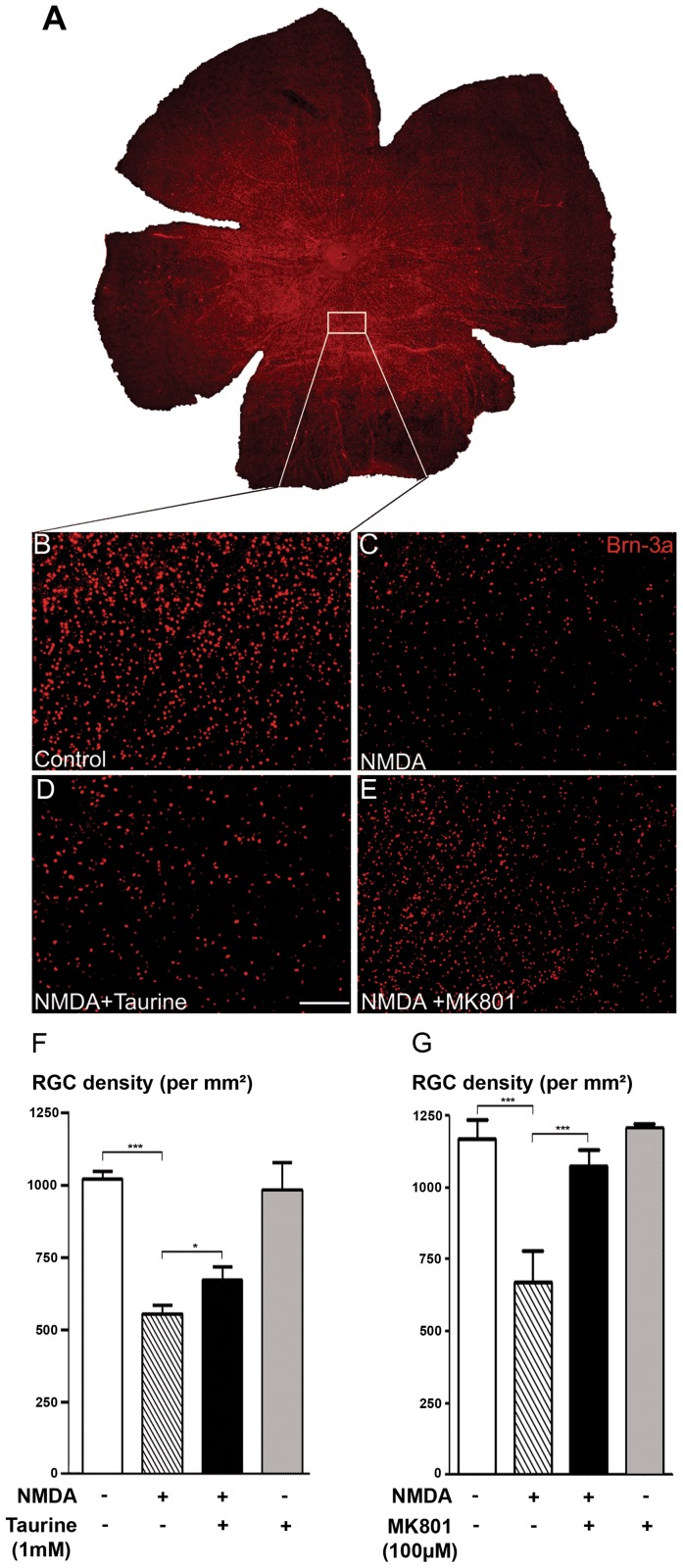
Taurine prevents the RGC death in NMDA-treated retinal explants. A) Digitalized reconstruction of a whole flat-mounted retinal explant immunolabeled with the POU4F1 (Brn3a) antibody. B–E) Representative enlarged fields from flat-mounted retinal explants acquired with the automated platform, showing POU4F1-immunopositive RGCs in a control untreated condition (Control; B) after 100 µM NMDA application (NMDA; C), after co-application of NMDA with taurine (1 mM; NMDA+Taurine; D) or after co-application of NMDA with MK801 (100 µM; NMDA+MK801; E) for 4 days. F) Quantification of RGC densities from whole flat-mounted retinal explants using the automated counting platform in Control group (n = 33, white bar); NMDA group: (n = 31, hatched bar); NMDA+Taurine group (n = 23, black bar) and Taurine group (n = 6, grey bar). G) Quantification of POU4F1-immunopositive RGC densities from whole flat-mounted explants under the control group (white bar), or the NMDA group (hatched bar), the NMDA plus MK 801 group (black bar), or finally the MK-801 group (grey bar) (n = 6 for each group). Data are expressed as means ± s.e.m. from n independent experiments. ***p<0.001, *p<0.05; One-way ANOVA followed by a Bonferroni post-hoc test. The scale bar represents 100 µm in panels (B–E).

Taurine was previously shown to prevent glutamate excitotoxicity in neurons [10], [11] and this mechanism is considered to be involved in different diseases with RGC degeneration such as glaucoma [21]. We therefore explored if taurine could also protect RGCs from glutamate excitotoxicity. Excitotoxicity was achieved by applying the glutamate receptor agonist NMDA (100 µM) for 4 days on rat retinal explants. At the end of the incubation, RGCs were immunolabeled with an anti-POU4F1 (Brn3a) antibody, a specific marker for RGCs. RGCs were counted on the whole flat-mounted retinal explants with an automated platform taking magnified images of explant areas (Fig. 3B) before reconstituting the whole explant image (Fig. 3A). We found that NMDA induced a drastic reduction (−44%) in RGC density (Control: 996.0±26.5 cells/mm^2^; NMDA: 555.6±31.8 cells/mm^2^; p<0.001, Fig. 3B–C, 3F). When taurine was added to the extracellular medium (1 mM), it significantly prevented part (25%) of this NMDA-elicited cell loss (NMDA+Taurine: 655.7±44.4 cells/mm^2^; p<0.05, Fig. 3D, 3F), whereas taurine applied alone in retinal explant did not modify the RGC density (Taurine: 959.2±91.5 cells/mm^2^; p>0.05, Fig. 3F). The excitotoxicity-induced RGC loss was specifically due to NMDA receptor stimulation since the NMDA receptor antagonist, MK-801 (100 µM) prevented 81% of the NMDA-induced RGC cell loss (n = 6 independent experiments; Fig 3E, 3G). Taurine can therefore partially prevent RGC losses from glutamate excitotoxicity.

### Taurine Prevents RGC Loss in Old Age DBA/2J Mice

**Figure 4:**
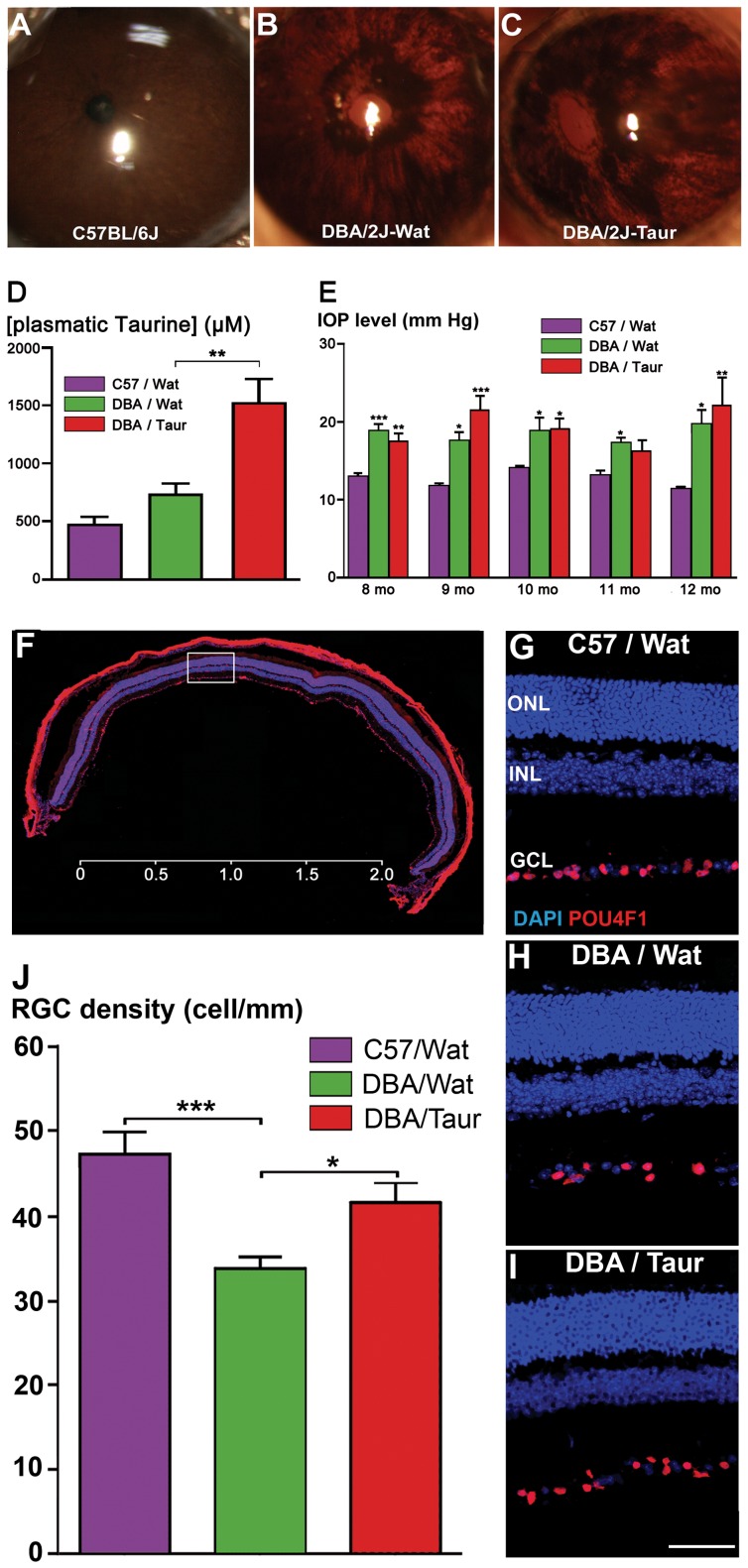
Taurine supplementation prevents RGC degeneration in DBA/2J mice, a genetic animal model of pigmentary glaucoma. A–C) Iris dispersion in DBA/2J mice. Normal iris in C57BL/6J mouse (A) and iris with pigment dispersion in DBA/2J mice drinking either taurine-free water (B) or taurine-supplemented (0.2 M) water (C) for 4 months. D) Taurine plasmatic levels in 12-month old C57BL/6J mice (C57/wat; purple bar) and DBA/2J mice drinking taurine-free water (DBA/Wat; green bar) or taurine-supplemented water (DBA/Taur; red bar). Data are means ± s.e.m. obtained from 8–10 animals per group. E) Intraocular pressure (IOP) levels measured at regular time intervals (8 to 12 months) on right eyes either from C57BL/6J mice (C57/wat; purple bars), from control DBA/2J mice (DBA/Wat; green bars) or from taurine-treated DBA/2J mice (DBA/Taur; red bars). Note the increased IOP in DBA/2J mice when compared to C57BL/6J mice but the lack of effect for the taurine supplementation on this increased IOP. Data (mm of Hg) are means ± s.e.m., n>10 animals for each group. F) Complete mouse retinal section viewed with a digital fluorescence scanner (Nanozoomer) showing POU4F1 (Brn3a) immunolabeling (red) and nuclear staining with DAPI (blue). G–I) Representative confocal images of retinal cryosections showing POU4F1-positive RGC immunolabeling (Brn3a; red) and retinal cell nuclei staining (DAPI; blue) in taurine-treated DBA/2J mice (DBA/Taur; H) and control DBA/2J drinking taurine-free water (DBA/Wat; H, I) as compared to C57BL6/J mice (C57/Wat; G). J) Quantification of RGC density on right eye retinal sections in C57BL/6J mice drinking taurine-free water (C57/Wat; purple bar; mean ± s.e.m, n = 6), in DBA/2J mice drinking either taurine-free water (DBA/Wat; green bar; mean ± s.e.m., n = 7) or taurine-supplemented water for 4 months (DBA/Taur; red bar; mean ± s.e.m., n = 6). ***p<0.001, **p<0.01 and *p<0.05, as compared to C57/Wat group in (E) or as compared to indicated groups in (D, J), One-way ANOVA followed by a Bonferroni post-hoc test. Scale bars represent 2 mm in pannel (F) and 50 µm in pannels (G–I).

To determine if taurine could affect RGC survival *in vivo*, taurine was supplemented to the drinking water of DBA/2J mouse, a validated genetic model of pigmentary glaucoma [22]. Indeed, old animals were showing an increase in intraocular pressure (IOP) compared to C57BL/6J mice, taken as control (see Fig. 4E). This IOP increase in old DBA/2J mice was due to iris atrophy and consecutive long-term pigment dispersion (Fig. 4A–B), thus sharing commonalities with pigmentary glaucoma, whereas C57BL/6J control mice did not present iris dispersion (Fig. 4A). Taurine supplementation (0.2 M, *per os* through the drinking water) was provided for 4 months, from 8- to 12-month old, when DBA/2J mice already exhibited a significant IOP increases, compared to control C57BL/6J mice (Fig. 4E). This supplementation in DBA/2J mice increased 2-fold the circulating plasma level of taurine (+106%; p<0.01, Fig. 4D), while no significant difference was found in the basal plasmatic taurine level between untreated DBA/2J and C57BL/6J mice (Fig. 4D). However, taurine treatment neither modified the pigment dispersion (Fig. 4B–C) nor significantly reduced IOP increases (Fig. 4E). To quantify RGC survival, retinal cryo-sections were prepared along the ventro-dorsal axis at 500 µm from the optic nerve and immunolabeled against POU4F1 (Brn3a) (Fig. 4F–I). POU4F1-positive RGC counting were performed blindly by two investigators on whole retinal sections, scanned at high magnification (Fig. 4F). At 12 month, the RGC density was reduced by 29% in DBA/2J mice when compared to C57BL/6J mice at the same age (C57BL/6J: 47.1±2.9 cells/mm; DBA/2J: 33.8±1.4 cells/mm, p<0.001; Fig. 4G–H, 4J). Interestingly, taurine supplementation from 8 to 12 months prevented 58% of this cell loss (DBA/2J + Taurine: 41.5±2.6 cells/mm, s.e.m., n = 6), the difference with the control DBA/2J group drinking taurine-free water was statistically significant (p<0.05, Fig. 4I, 4J). No clear modification of Tau-T immunolabeling was observed in DBA/2J mice as compared to C57BL6/2J in the ganglion cell layer (data not shown). These results indicate that taurine supplementation partly prevents RGC degeneration due to an elevated IOP in DBA2/J mice.

### Taurine Prevents RGC Death in Rat Subjected to Episcleral Vein Cauterization

**Figure 5:**
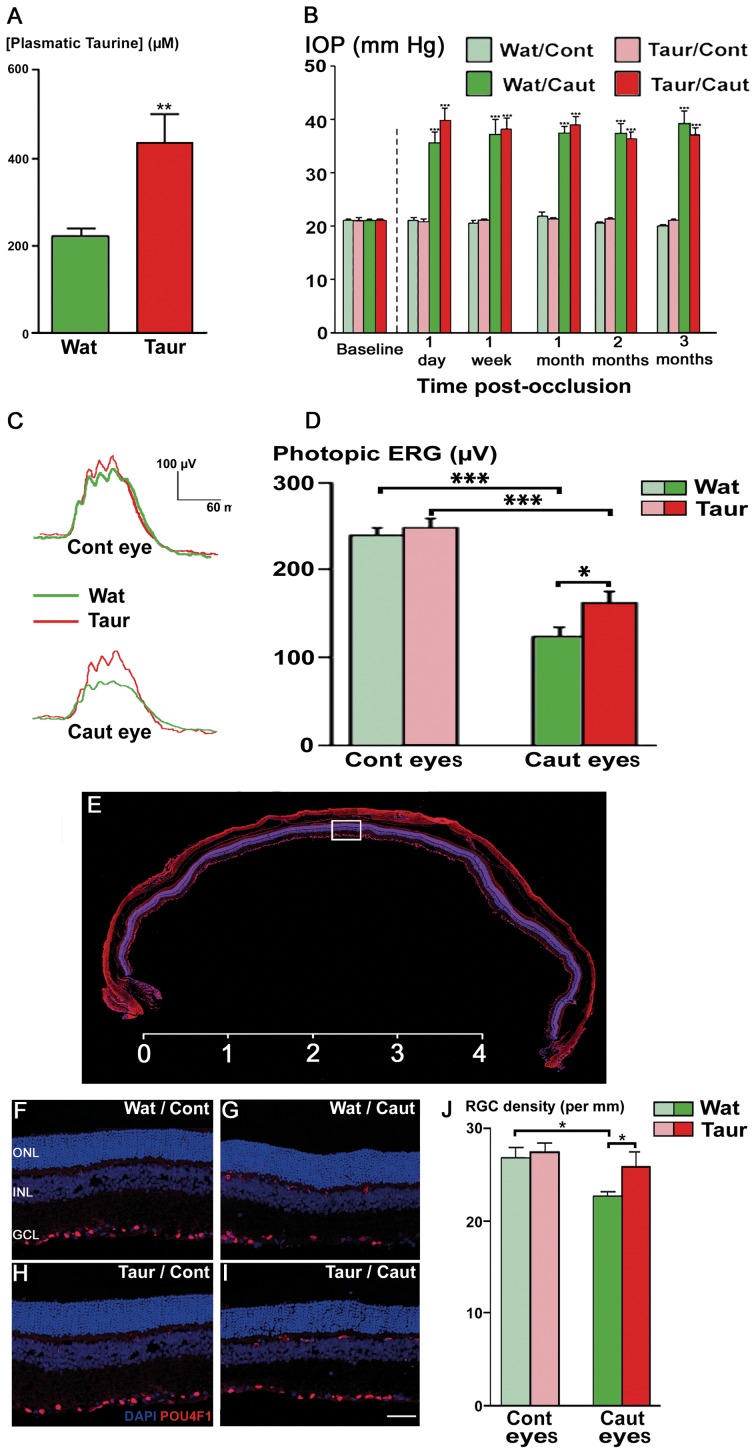
Taurine supplementation prevents RGC degeneration in glaucomatous Long-Evans rats following episcleral vein occlusion. A) Taurine plasmatic levels in Long-Evans rats drinking taurine-free water (Wat; green bar; mean ± s.e.m., n = 21) or taurine-supplemented water for 3 months (Taur; red bar; mean ± s.e.m., n = 24; **p<0.01, student’s t-test). B) Intraocular pressure (IOP) levels measured at regular time intervals after episcleral vein occlusions by cauterization on the operated right eyes (Caut) and unoperated left eyes (Cont) in rats drinking taurine-free water (Wat, green bars) or in taurine-treated rats (Taur; red bars). The difference between the IOP remained statically significant between the operated and unoperated eyes in both animal groups throughout the study (mean ± s.e.m., n>14 for all groups; ***p<0.001 as compared to water/control eyes, one-way ANOVA followed by a Bonferroni post-hoc test). C) Representative photopic electroretinograms (ERG) recorded from an operated right eye (Caut eye) and an unoperated left control eye (Cont eye) after 3 months of episcleral vein occlusion in taurine-treated rats (Taur, red traces) and in control rats drinking taurine-free water (Wat; green traces). D) Quantification of photopic ERG amplitudes measured from the left unoperated eyes (Cont eyes) and right operated eyes (Caut eyes) in taurine-supplemented rats (Taur; red bars; mean ± s.e.m., n = 24) or control rats drinking taurine-free water (Wat; green bars; mean ± s.e.m., n = 32 for control eyes and 40 for cauterized eyes) after 3 months of vein occlusion. E) Complete rat retinal section viewed with a digital fluorescence scanner (Nanozoomer) showing POU4F1 (Brn3a) immunolabeling (red) and nuclear staining with DAPI (blue). F-I) Representative confocal images of retinal cryo-sections showing the POU4F1 (Brn3a) positive RGC immunolabeling (red) and cell nuclei staining (DAPI; blue) performed in unoperated left eyes (Cont; F,H) and cauterized right eyes (Caut; G,I) in rats without (Wat; F, G) or with taurine supplementation (Taur; H, I) added to their drinking water. J) Quantification of POU4F1 (Brn3a) positive RCG densities in both unoperated left (Cont eye) and operated right (Caut eye) eyes from rats without (Wat; green bars; mean ± s.e.m., n = 11 and 10 for control and cauterized eyes, respectively) or with taurine supplementation (Taur; red bars; mean ± s.e.m., n = 11 and 9 for control and cauterized eyes, respectively). *p<0.05 and ***p<0.001 as compared to indicated group in (B, D, J), one-way ANOVA followed by a Bonferroni post-hoc test. Scale bars represent 4 mm in panel (E) and 50 µm in panels (F-I).

To further confirm the potential preventive role of taurine supplementation against RGC degeneration *in vivo*, we investigated its effect in another classic animal model for glaucoma: the episcleral vein occlusion in rats [23]. Indeed, the occlusion of three episcleral veins by cauterization generates a long-term (3 months at least) and stable increase in IOP from the operated right eye as compared to the unoperated left eye, taken as control (p<0.001; Fig. 5B). The taurine supplementation (0.2 M for 3 months) administered in the drinking water increased significantly (by 95%) the plasma taurine concentration compared to control rats drinking taurine-free water (p<0.01; Fig. 5A). However, this increase in the plasma taurine concentration did not affect the IOP increase measured in the operated eye over the treatment period (Fig. 5B). Three months after the occlusion of the episcleral veins, the operated eye showed a significant reduction in the amplitude of the photopic electroretinogram (ERG) (p<0.001; Fig. 5C–D). The taurine supplementation prevented partially, but significantly, this functional decrease (p<0.05; Fig. 5C–D). These changes in photopic ERGs observed on cauterized eyes were not due to either cone degeneration or cone rescue because the quantification of cone-arrestin positive cells revealed no change in cone density among the different experimental groups (data not shown). RGC numbers were then quantified blindly by 2 different investigators on cryosections prepared along the ventro-dorsal axis and immunolabeled against POU4F1 (Brn3a) (Fig. 5E–J). The IOP increase induced a significant 15% RGC loss in operated right eyes (22.7±0.5 RGC/mm) when compared to unoperated left eyes (26.8±1.2 RGC/mm, p<0.05; Fig. 5F–G, J). Taurine supplementation significantly prevented 87% of this RGC loss in the operated right eyes (26.3±1.5 RGC/mm; p<0.05; Fig. 5I, J), whereas no effect was observed in the unoperated left eyes (27.8±0.8 RGC/mm; Fig. 5H, J). These results indicate that taurine supplementation can prevent RGC loss in this animal model of RGC degeneration.

### Taurine Reduces the Secondary RGC Degeneration Following Photoreceptor Loss in P23H Rats

**Figure 6:**
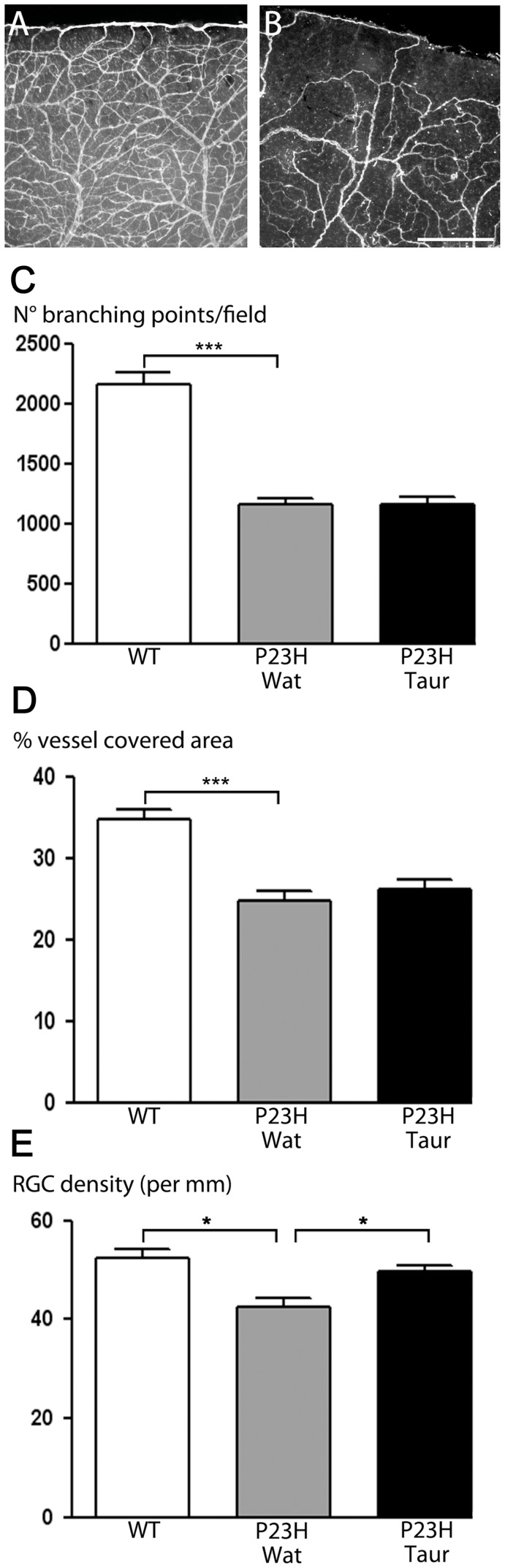
Taurine prevents RGC degeneration in P23H rats with blood vessel atrophy. A, B) Representative pictures showing the lectin staining of blood vessels at the periphery of flat-mounted retinae from a one-year old Sprague-Dawley rat (A) and a one-year old heterozygous P23H rat (B). C,D) Quantification of number of blood vessel branching points (C) and percentage of blood vessel coverage (D) obtained on the whole retina by an automated platform in Sprague-Dawley wild-type animals (WT; white bar), in untreated heterozygous P23H rats (P23H Wat; grey bar) and in taurine-supplemented P23H rats (P23H Taur; black bar). These quantifications demonstrated the absence of taurine effect on blood vessel atrophy (means ± s.e.m., n = 5 for each group). E) Quantification of POU4F1 (Brn3a) immunopositive RGCs in retinal cryosections from Sprague-Dawley wild-type animals (WT, white bar), from untreated heterozygous P23H rats (P23H Wat; grey bar) and from taurine supplemented P23H rats (P23H Taur; black bar). Data expressed as RGC per mm of retinal section, are means ± s.e.m. from n = 7 animals for each group. ***p<0.001 and *p<0.05 as compared to indicated groups, one-way ANOVA followed by a Bonferroni post-hoc test. Scale bar represents 100 µm in panels (A–B).

To investigate if a taurine depletion could also partly prevent the reported secondary RGC degeneration in *Retinitis pigmentosa* [24], we administered taurine to P23H rats, a model of this disease [25]. Heterozygous P23H rats (line 1) were given free access to drinking water containing taurine (0.1 M) for three months from 9- to 12-month old, an age when most photoreceptors have already degenerated [26]. As a vascular atrophy is also occurring during photoreceptor degeneration [25], we first verified that the taurine treatment was not affecting this process. At the end of the treatment, the vascular atrophy (branching points, blood vessel coverage) was clearly observed in P23H rats when compared to age-matched Sprague-Dawley rats, but this atrophy was not modified by the taurine supplementation (Fig. 6A–D). At one year, P23H rats exhibited a significant 18% loss in RGC density (42.6±4.7 cells/mm), when compared to the age-matched Sprague-Dawley control rats (52.3±5.9 cells/mm) (Fig. 6E). Three-month taurine supplementation from 9- to 12-month old prevented significantly 65% of this RGC loss (49.0±2.9 cells/mm; p<0.05; Fig 5E). These results indicate that taurine can also prevent RGC loss in this animal model of *Retinitis pigmentosa*.

## Discussion

Our previous experiments on vigabatrin-treated animals had suggested that taurine depletion can cause RGC degeneration [18]. Here, we provide evidence that taurine can also promote the survival of adult RGCs in a pure culture. This result demonstrates a direct neuroprotective action of taurine on RGCs, which is consistent with the taurine-elicited resistance to hypoxia in an immortalized RGC cell line [20]. The specific expression of the taurine transporter in freshly purified rat RGCs (not cultured) suggests that an intracellular mechanism is involved in taurine protective action. This hypothesis was validated here by the loss of taurine neuroprotection found in presence of the taurine transporter inhibitor, GES. As reported in the immortalized RGC cell line, the taurine neuroprotection of RGCs could occur by reducing the intracellular calcium levels and by inhibiting the opening of mitochondrial permeability transition pores [20]. Taurine was also shown to be essential for the translation of mitochondrial DNA [27]. This effect on mitochondrial activity could be crucial for RGC survival because mitochondrial dysfunction is often leading to the selective RGC degeneration in diseases like optic neuropathies [28]. The *in vitro* survival effect reported in this study was obtained at a millimolar concentration that remains in the physiological range since the retinal concentration of taurine was reported as high as 50 mM [8]. The taurine concentration used in our experiments is also similar to those used for *in vitro* studies on cerebellar neurons [11] and on the immortalized RGC line [20]. Taken together, these results indicate that taurine can directly stimulate RGC survival through an intracellular mechanism following its uptake by the taurine transporter. This conclusion is consistent with the RGC degeneration observed in animals when administering a Tau-T blocker although the blocker had also a systemic effect reducing the circulating level of taurine [29]. Similarly, the circulating level of taurine was reduced in Tau-T knock-out mice but RGC degeneration was not investigated; it was only reported that the RGC layer appeared to be intact, but may have fewer cells than in wild type mice [30].

Taurine was previously reported to prevent glutamate excitotoxicity in embryonic cultured cerebellar neurons [11] or mixed brain neurons [10]. Our present study extends this conclusion to adult RGCs in NMDA-treated retinal explants. The intracellular effects of taurine discussed above could explain this prevention of RGC glutamate excitotoxicity in NMDA-treated retinal explants. Especially, the taurine-induced reduction of intracellular Ca^2+^ levels, as described in brain neurons [10], [11] and in immortalized RGCs under hypoxia [20], could limit the toxic Ca^2+^ overload resulting from the prolonged activation of the Ca^2+^-permeable NMDA receptors. The taurine-mediated reduction in glutamate excitotoxicity could also be attributed to the taurine effect on glutamate uptake [31]. Despite the negative conclusion of the clinical trial with memantine, a blocker of NMDA receptors [32], glutamate excitotoxicity is considered as an important molecular mechanism in RGC degeneration during glaucoma and other retinal diseases with an ischemic contribution [21]. Therefore, the protective effect of taurine on pure RGC cultures and on RGC glutamate excitotoxicity could explain the *in vivo* RGC rescue in glaucoma animal models.

The observed RGC neuroprotection by taurine supplementation in the animal models suggests that the retinal taurine content may decrease below the optimum level for RGC survival. In fact, a lower ocular perfusion pressure, which was defined as a risk factor for primary open angle glaucoma at the 6^th^ consensus meeting of the World Glaucoma Association [33], could cause a decrease in the retinal taurine content. Indeed, the reduction in the ocular perfusion pressure in open angle glaucoma is associated to an impaired ocular blood flow [33], which will likely alter the high taurine uptake index through the blood-retinal barrier [34] via taurine transporters expressed by retinal capillary endothelial cells [35]. This hypothesis is supported by a previous report on glaucomatous dogs showing a taurine depletion in their damaged photoreceptors [36]. Taurine depletion could thus explain both the RGC loss and the photoreceptor degeneration although the later photoreceptor degeneration is very limited in glaucomatous patients as in glaucomatous monkeys [37]. In fact, photoreceptor degeneration has been a matter of controversy in the field of glaucoma [37], [38] and it may be restricted to secondary glaucoma with reduced choroidal perfusion [39]. In other types of glaucoma, the taurine depletion may be restricted to the inner retina because their IOP increase does not affect the blood flow at the choroid level but that of the central retinal artery [40], [41]. In addition, human RGCs could be more sensitive than photoreceptors to a reduced taurine retinal content as indicated by their primary degeneration in vigabatrin-treated patients [14]–[17].

In order to further demonstrate the potential of taurine supplementation in preventing RGC degeneration, we have used an animal model of *Retinitis pigmentosa*, the P23H rat, which exhibits RGC degeneration secondary to photoreceptor loss. This secondary RGC degeneration had been attributed to the compression of RGC axons by intra-retinal vessel contraction [42]. A major vascular atrophy and remodeling is indeed observed during, and following photoreceptor degeneration [25]. Interestingly, we demonstrated that taurine administration prevented the RGC degeneration. This result suggests that the vascular atrophy could also contribute to the RGC degeneration by reducing the number of capillary endothelial cells transporting taurine into the retinal tissue [35]. Consistently, the retinal content of taurine was reported to decrease during photoreceptor degeneration in animal models of *Retinitis pigmentosa* [8]. Altogether these results suggest that reducing any parameter affecting taurine retinal influx may lead to RGC degeneration: (i) taurine plasma concentration as in the course of vigabatrin-treatments [18], (ii) ocular blood flow as in glaucoma [33], [40], [41] and (iii) density of the retinal vascular plexus as in *Retinitis pigmentosa* [25].

Oxidative stress appears as another major mechanism in RGC degeneration [43]. Different anti-oxidants were already proposed for prevention of RGC degeneration, because they were found to be decreased in glaucomatous patients. For instance, the level of sulfhydryl groups (glutathione) was found significantly lowered in the aqueous humour of patients with open-angle glaucoma, particularly in those with disease Stages II and III [44]. Although taurine is part of the sulfur metabolism, there is no data concerning the ocular taurine content in glaucomatous patients. But interestingly, diabetic patients exhibiting a RGC degeneration during diabetic retinopathy [45] were reported to show a decrease in plasma taurine [46]; vigabatrin-treated patients have similar features: a decrease in plasma taurine concentration [13] and a RGC loss [14]–[17]. Future studies will have to define whether taurine is a crucial anti-oxidant molecule depleted in the retina during glaucoma and whether its supplementation can slow down the degenerative process. In the case of *Retinitis pigmentosa*, a combination treatment including taurine was already found to improve patient vision although this effect was attributed to an improvement in photoreceptor function [9]. However, an effect on RGCs could also contribute to the preservation of the visual field in this clinical study.

In all our animal experiments, the taurine supplementation increased by 2 fold the plasma taurine concentration although the starting concentration was different in mice and rats. Future studies will have to investigate how the retinal taurine concentration is related to both this circulating plasma concentration and the transporter expression level in retinal endothelial cells. In humans, taurine supplementation has already been considered for the treatment of various diseases and no human toxicity or undesirable side effects have been reported even at doses as high as 6 g/day [47]. In fact, low doses (1.5 g/day) were already found to increase more than 2-fold the taurine plasma concentration in human subjects [48], an increase shown to prevent the RGC degeneration in our different animal models. This supplementation corresponds to approximately a 7-fold increase in the average daily nutritional taurine intake (216 mg/day; [49]). Future epidemiological studies are needed to investigate how the nutritional taurine intake is correlated to the development of RGC degenerative diseases. Indeed, a great variability in nutritional taurine intake was reported among different world populations when correlating taurine levels with heart failure [50]. These considerations indicate that a proper diet or taurine supplementation could contribute to the prevention and/or treatment of RGC degeneration in different pathological conditions.

## Materials and Methods

### Animals

DBA/2J mice were obtained from Charles River (L’Arbresle, France). Long-Evans rats, used for RGC pure cultures, retinal explants and vein occlusion experiments, were purchased from Janvier (Le Genest Saint-Isle, France). Heterozygous P23H rats were generated from a backcross between homozygous transgenic P23H mutant rats (line 1, kindly provided by Dr Lavail) and Sprague–Dawley rats, considered as controls. Sprague–Dawley rats were purchased from Charles River. Animals were housed with a 12 h dark/light cycle with food and water available *ad libitum*. All Experiments have been carried out in accordance with the European Community Council Directives (86/609/EEC) and with the ARVO (Association for Research in Vision and Ophthalmology) statement for the Use of animals in ophthalmic and visual Research.

### Purification and Culture of Retinal Ganglion Cells (RGCs)

RGCs were isolated from retinae of adult Long-Evans rat (8-week old) with an immunopanning technique, according to protocols previously described in young rats [51] and adult animals [52]. Purified adult RGCs were seeded at initial density of 2×10^4^ cells/well, in a 48-well plates, containing 8 mm in diameter coverslips previously coated successively with poly-D-lysine and laminin, (2 µg/cm^2^ for 45 min and 1 µg/cm^2^ overnight, respectively). RGCs were cultured in a low nutritive medium (control) constituted by Neurobasal-A (Invitrogen, Carlsbad, CA, USA) supplemented with 2 mM L-glutamine (Sigma-Aldrich, St-Louis, MO, USA) and kept in a humidified chamber at 37°C containing 5% CO_2_ for 6 days *in vitro* (DIV). Taurine (1 mM; Sigma-Aldrich), GuanidinoEthane Sulfonate (GES; Toronto Research Chemical, Toronto, Canada) or B27 (50X; Invitrogen) were applied in the culture medium for the whole culture period.

### Immunocytochemistry

Immunolabeling experiments were performed on RGC pure cultures. Briefly, cells were fixed 15 min at 4°C with paraformaldehyde (4%; Sigma-Aldrich) and stained with antibodies anti-NF-200 (rabbit polyclonal; dilution 1∶500, Sigma-Aldrich), anti βIII-tubulin (Mouse monoclonal, dilution 1∶500; Sigma-Aldrich) or anti-taurine transporter1 (Tau-T) (rabbit polyclonal, dilution 1∶100; Euromedex, Strasbourg, France). Because RGC purification by immunopanning used the THY-1 antibody, microglial cells were also stained using a mouse anti-Cd11b/c monoclonal antibody (dilution 1∶500; BD Pharmigen, San-Diego, CA, USA). Counterstaining with 4′,6-Diamidino-2-phenylindole (DAPI; 10 µg/mL; Sigma-Aldrich) was performed to label cell nuclei and DAPI-positive cells were counted from 10 field per well, and reported to number of NF-200 or βIII-tubulin positive cells to evaluate RGC purity.

### Viable RGC Counting

After 6 DIV, remaining viable cells were revealed by a calceinAM labeling (Invitrogen) while dead cells were stained by ethidium homodimer-1. The dyes were diluted in Phosphate Buffer Saline (PBS) medium and incubated for one hour in incubator (humidified chamber, 37°C, 5% CO_2_). Images were acquired under an epifluorescence microscope for seven fields at fixed and identical positions for all coverslips (Leica DM 5000B, Solms, Germany). Viable cells were then quantified by two persons not aware of the experimental groups. In each experiment, cell survivals were normalized to the control group, prior to averaging results from the different experiments.

### RT-PCR and qPCR Analysis

RNA was extracted from freshly purified RGCs (not cultured), full retina, liver or kidney of Long-Evans rats, using the RNeasy plus mini kit (Qiagen, Courtaboeuf, France). Reverse transcription were then made using the SuperScript II (Invitrogen) incubation firstly at room temperature for 10 min, then at 42°C for 1 hour and finally at 70°C for 15 min in presence of Random Hexamer (25 ng/µl; Promega, Madison, WI, USA), dNTP (500 µM; Invitrogen), RNasine (2 U/µl; Promega). The resulting cDNA was purified with phenol/chlorophorme/isoamyl mixture, precipitated with ethanol and suspended in Tris/EDTA solution (10 mM/1 mM; pH = 8.0) and finally stored at −80°C.

PCR experiments were conducted from purified cDNAs, in the presence of 200 nM of each primer (forward and reverse) for each gene amplified, 200 µM dNTP (Invitrogen), 1.5 mM MgCl_2_ and 1.0 U Taq DNA polymerase (Invitrogen). Primer sequence for each gene, are described in Table 1. Amplification was performed for 40 cycles (30 sec at 95°C; 45 sec at 60°C and 1 min at 72°C) with the thermocycler “iCycler” (Biorad, Marnes-la-coquette, France) and PCR products were separated by electrophoresis in 2% agarose gel, stained by ethidium bromide.

**Table 1:** Primer sequences used in PCR and qPCR experiments.

Name	Sequence	Specie	Sequence length (bp)	Size of amplicon
GAPDH-F	CGATCCCGCTAACATCAAAT	rats	20	324
GAPDH-R	CCACAGTCTTCTGAGTGGCA		20	
Tau-T-F	TCCTGGAGGTCATCATAGGC	rat	20	300
Tau-T-R	TCACAGGCGAAGTGAAGTTG		20	
Brn-3a-F	AGGCCTATTTTGCCGTACAA	rat	20	155
Brn-3a-R	CGTCTCACACCCTCCTCAGT		20	
Rhodopsin-F-	TACATGTTCGTGGTCCACTT	rat	20	230
Rhodopsin-R	TTGGAGCCCTGGTGGGTAAA		20	

Primers were used for the amplification of Glyceraldehyde 3-phosphate deshydrogenase (GAPDH), Taurine transporter (Tau-T), Brn-3a and Rhodopsin. For each gene a forward (F) and reverse (R) primers were used.

Quantitative PCR (qPCR) was performed using the Applied Biosystem Fast 7500 (Applied Biosystems, Forster City, CA, USA). Briefly, reaction were made using MicroAmp Fast optical 96-well plate, in a 20 µl final volume, containing 2 µl cDNAs (12.4ng/well), 10 µl Power SYBR® Green PCR Master Mix (2X, Invitrogen), 1 µl of each primer (500 nM for each) and 6 µl of RNAse/DNAse free water distilled (Invitrogen). For each gene, protocol was constituted by an initial denaturation step (95°C for 10 min), followed by an amplification step (95°C, 15 sec and 60°C, 1 min for 45 cycles), a fusion step (1 cycle of 95°C, 15 sec; 60°C, 1 min; 95°C, 15 sec and 60°C, 15 sec). All samples were run in duplicates and the fluorescent threshold values (Ct) was determined using the 7500 system software.

### NMDA-treated Retinal Explants

Retinal explants from adult rats were cultured similarly as described previously [53]. Briefly, retinae were dissected and flattened on a polycarbonate membrane inserts (Transwell Costar, Sigma-Aldrich). Retinal explants were cultured for 4 DIV in Neurobasal-A medium (Invitrogen) supplemented with 2 mM glutamine (Sigma-Aldrich) and B27 (50X, Invitrogen). RGCs were subjected to excitotoxicity by addition of N-Methyl-D-aspartic acid (NMDA, 100 µM, Sigma-Aldrich) into the culture medium during all the time of culture. Taurine (1 mM) was incubated into the medium 1 hour before NMDA application and let for the whole culture period (4 DIV). RGC immunostaining was then performed on flat-mounted retinal explants as follow the procedure described by García-Ayuso et al [54] using a mouse anti-Pou4F1 (Brn-3a) monoclonal antibody (dilution 1∶500; Millipore Corporation, Billerica, MA, USA). POU4F1-immunopositive RGCs were then counted automatically using Metamorph software (Ropert scientific, Evry, France) on the whole flat-mounted retinal explants.

### *In vivo* Experiments

Taurine was administered *per os* by its addition into the drinking water for 4 months from 8- to 12–month old in DBA/2J mice, a genetic animal model for pigmentary glaucoma [22]; for 3 months in 2-month old Long-Evans rats with episcleral vein occlusion, an evoked glaucoma animal model; and for 3 months from 9 to 12 months of age in P23H heterozygous rats, a genetic model of R*etinitis pigmentosa*. Visualization of iris dispersion was examined in awake DBA/2J mice using a slit lamp (DC-3, Topcon, Clichy, France). Occlusion of episcleral veins was performed on 8-week old Long-Evans rats as previously described [23]. Three episcleral veins were cauterized only on the right eye, using bipolar forceps. Photopic ERGs were recorded using two gold loop electrodes with light stimuli (10 cds/m^2^) applied on a light background (25 cd/m^2^, adaptation time 5 min), as previously described [13]. IOP was measured in the two glaucoma models using a tonometer (Tonolab, Icare, Helsinki, Finland). Plasmatic taurine measurements were performed on each animal by Serba laboratories (Cergy-Pontoise, France) using HPLC technique.

### RGC and Vessel Quantification

Immunostaining were performed on cryosections from rats or mice eye-cups using the anti-Brn-3a monoclonal antibody (dilution 1∶100, Millipore). RGC quantification relied on POU4F1 (Brn3a) immunolabeling because a strong correlation was reported between POU4F1-immunopositive RGCs and retrogradely dye-stained RGCs, as early as 3 weeks after the initial RGC lesion [55]. In addition, POU4F1-immunopositive RGCs represent the majority of the RGC population (more than 75%) [56]. POU4F1/DAPI-positive RGCs were counted from the whole vertical sections scanned with a Digital Pathology System: Nanozoomer (Hamamatsu, Massy, France) by two independent investigators unaware of the experimental groups. To evaluate the RGC density, the RCG numbers were normalized to the length of the retinal section measured with NDP-view software (Hamamatsu).

Blood vessels were labeled from retinal flatmounts of P23H rats (and their control) using a lectin from *Bandeiraea simplicifolia* (Sigma-Aldrich). Full retinae were digitalized to automatically quantify (i) the percentage of blood vessel coverage on the total retina and (ii) the number of vessel branching points using MetaMorph software.

### Statistical Analysis

All data are expressed as means ± s.e.m. Gaussian distribution of raw data was tested with Shapiro-Wilk normality test. A two-tailed unpaired Student’s t-test was used to compare means of 2 groups. For more than 2 groups compared, a one-way ANOVA was used for variance analysis, followed in case of significance by either a Bonferroni post-hoc test (Gaussian distribution) or a Dunns post-hoc test (no Gaussian distribution) to compare the means of each group. Differences were considered significant at *p<0.05, **p<0.01 and ***p<0.001.
